# A Novel Rectal Propranolol Strategy in Neonatal Supraventricular Tachycardia Complicated by Surgical Necrotizing Enterocolitis

**DOI:** 10.1055/a-2942-1294

**Published:** 2026-09-09

**Authors:** M. Siddharth, Ashishkumar Banpurkar, Abhijit Benare, Anusha Rao, Umesh Vaidya, Komal Bijarniya, Manali Bhuyan, Bhakti Dhamangaonkar

**Affiliations:** 1 Department of Neonatology 722952 Ankura Hospital for Women and ChildrenPuneMaharashtraIndia; 2 Department of Paediatric Cardiology 722952 Ankura Hospital for Women and ChildrenPuneMaharashtraIndia; 3 Department of Pediatric SurgeryAnkura Hospital for Women and ChildrenPuneMaharashtraIndia; 4 Department of Obstetrics and GynaecologyAnkura Hospital for Women and ChildrenPuneMaharashtraIndia

**Keywords:** necrotizing enterocolitis, supraventricular tachycardia, propranolol, novel therapy, neonate, surgical NEC

## Abstract

**Objective** Surgical necrotizing enterocolitis (NEC, Bell stage 3B) complicating neonatal supraventricular tachycardia (SVT) is exceedingly rare. We describe a novel per-rectal propranolol strategy to maintain antiarrhythmic prophylaxis during mandatory nil per oral (NPO) status imposed by NEC.

**Study Design** This study includes a single case report at a tertiary neonatal unit in Western India.

**Results** A 35-week neonate with antenatal fetal tachyarrhythmia (>300 beats per minute [bpm]) developed postnatal SVT at 6 h of life (280–300 bpm), requiring adenosine cardioversion and oral propranolol. On day of life (DOL) 4, hematochezia, abdominal distension, and portal venous gas indicated Bell stage 3A NEC, mandating NPO status. With enteral routes contraindicated, propranolol was continued per rectum, to our knowledge the first reported neonatal antiarrhythmic use of this route. Deterioration to pneumoperitoneum by DOL 8 necessitated laparotomy with jejuno–jejunal and ileo–caecal resection and anastomosis. No SVT recurred during NPO. The infant was discharged on DOL 25 on oral propranolol.

**Conclusion** This first Indian report of SVT-associated surgical NEC highlights two novelties: Bell stage 3B NEC requiring surgery and per-rectal propranolol as a novel antiarrhythmic bridge during NPO. Clinicians should maintain vigilance for NEC in neonates with SVT regardless of hemodynamic stability, and consider the rectal route for propranolol when NPO.

## Introduction

Necrotizing enterocolitis (NEC) is the most common and devastating gastrointestinal emergency encountered in the neonatal intensive care unit (NICU), with a mortality of 15 to 30% among affected preterm infants. 1 Its pathogenesis is multifactorial, and intestinal ischemia is considered a central, unifying mechanism. 2 Supraventricular tachycardia (SVT) is the most common pathological tachyarrhythmia in neonates, with an estimated incidence of 1 in 250 to 1 in 1000 children, and is typically managed with adenosine cardioversion followed by β-blocker prophylaxis. 3 4 NEC is staged according to the original Bell et al criteria. 5

The association between SVT and NEC was first described in 1995 by Cale and Speidel, and since then, approximately ten additional cases have been reported in the literature. 6 7 8 9 10 11 12 13 14 15 The proposed pathophysiological mechanism involves SVT-induced mesenteric hypoperfusion through a “steal phenomenon,” reduced diastolic coronary and splanchnic filling, and subsequent reperfusion injury upon rhythm restoration. 10 11 Despite this mechanistic plausibility, no controlled study has confirmed a statistically significant association. 12

Cases of surgical NEC (Bell stage 3B) specifically in the context of neonatal SVT are exceptionally rare, with most published reports describing medical NEC managed conservatively. Additionally, the literature largely originates from high-income countries, with no reports to date from the Indian subcontinent. We present a late preterm neonate from Pune, India, in whom antenatal-onset SVT was followed by surgical stage 3B NEC requiring laparotomy, with histopathological confirmation of ischemic bowel injury.

## Case Presentation

A male infant was delivered by emergency lower-segment caesarean section at 35 weeks of gestation to a 31-year-old primigravida mother. The mother presented in active labor with a previous uterine scar, clinical scar tenderness, a nonreassuring nonstress test, and radiological evidence of uterine scar thinning on obstetric ultrasonography. Fetal tachyarrhythmia with a heart rate exceeding 300 beats per minute (bpm) was identified incidentally on the preoperative scan. The decision to proceed to emergency LSCS was therefore driven by multiple concurrent obstetric indications; the fetal tachyarrhythmia represented an additional finding rather than the sole indication for delivery. No fetal hydrops or echocardiographic features of congestive cardiac failure were identified on any prior or current antenatal scan, suggesting a likely short-duration arrhythmia. The infant was born with a birth weight of 2740 g (appropriate for gestational age), with Apgar scores of 8 and 9 at 1 and 5 min, respectively. No maternal cardiac, metabolic, or autoimmune history was documented. The infant cried immediately at birth but was transferred to the NICU owing to prematurity and early respiratory distress. The infant was born with a birth weight of 2740 g (appropriate for gestational age), with Apgar scores of 8 and 9 at 1 and 5 min, respectively. No maternal cardiac, metabolic, or autoimmune history was documented. The infant cried immediately at birth but was transferred to the NICU owing to prematurity and early respiratory distress.

On admission to the NICU, the infant had a heart rate of 140 bpm with an oxygen saturation of 98% on room air. The infant was commenced on noninvasive ventilation for respiratory distress syndrome, with a chest radiograph and clinical findings consistent with this diagnosis. At 6 h of life, the infant developed a sustained tachyarrhythmia at 280–300 bpm with a narrow QRS complex and absent P-waves on electrocardiogram, consistent with SVT without features of Wolff–Parkinson–White syndrome. Four to five discrete SVT episodes occurred, each requiring intravenous adenosine (starting at 0.1 mg/kg, escalating doses up to 0.3 mg/kg) for successful cardioversion. Pediatric cardiology consultation was obtained, and a two-dimensional echocardiogram demonstrated an atrial septal defect (ostium secundum) with otherwise structurally normal cardiac anatomy and good biventricular function. Propranolol was commenced at 2 mg/kg/day and subsequently uptitrated to 4 mg/kg/day following breakthrough SVT on day of life (DOL) 3.

**Fig. 1:**
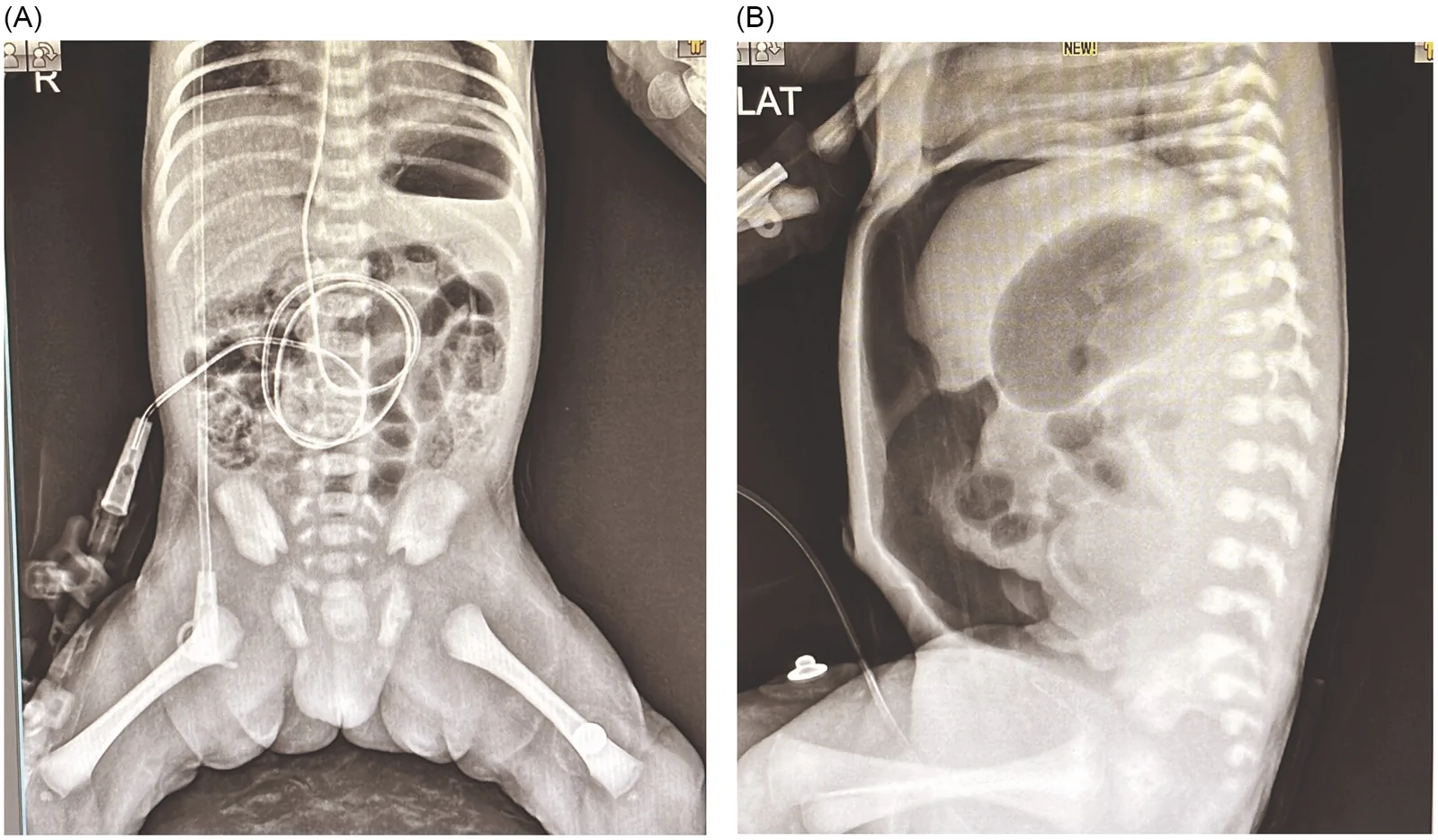
Abdominal radiographs demonstrating the radiological progression of necrotizing enterocolitis (NEC). ( **A** ) Anteroposterior erect abdominal radiograph on day of life (DOL) 4 demonstrating dilated bowel loops with pneumatosis intestinalis and portal venous gas. Bell stage 3A NEC was diagnosed on the basis of combined radiographic and abdominal ultrasonographic findings (the latter demonstrating free intraperitoneal fluid/ascites), together with the clinical presentation; An umbilical venous catheter is noted in situ. ( **B** ) Lateral erect abdominal radiograph on DOL 8 demonstrating a large crescentic anterior lucency representing free intraperitoneal air, upgrading the diagnosis to Bell stage 3B NEC and prompting surgical intervention.

Minimal enteral feeding via orogastric tube was initiated on DOL 4 since the SVT episodes had become less frequent and the infant had been free from episodes for nearly 24 h. On DOL 4, within 12 h of commencement of minimal enteral nutrition (MEN) and gradual feed escalation as per unit policy, the infant developed hematochezia and increasing abdominal distension with clinical deterioration including poor perfusion, requiring fluid resuscitation, inotropic support (noradrenaline and dobutamine), and hydrocortisone for refractory shock. The infant was intubated and commenced on pressure-controlled mechanical ventilation. An abdominal radiograph demonstrated pneumatosis intestinalis with portal venous gas ( Fig. 1A ). Abdominal ultrasonography confirmed free intraperitoneal fluid (ascites), consistent with Bell stage 3A NEC. Enteral feeds were discontinued, TPN was initiated, and broad-spectrum antibiotics (piperacillin–tazobactam) were commenced. With the infant now nil per oral (NPO) for NEC, continued arrhythmia prophylaxis presented a significant challenge. Intravenous β-blocker therapy was considered; however, IV propranolol carries a recognized risk of severe hypotension. Accordingly, propranolol was administered via the per-rectal route, using the existing oral liquid preparation (tablet crushed and diluted in normal saline) administered three times daily at a dose of 1 mg/kg per dose (total 3 mg/kg/day). This dose was intentionally reduced from the 4 mg/kg/day that had been reached orally prior to NEC onset, as a precautionary measure given the concurrent hemodynamic instability and inotrope dependence. Continuous electrocardiographic and intra-arterial blood pressure monitoring were maintained throughout the NPO period, alongside regular blood glucose surveillance as part of standard critical care. This strategy was clinically effective: no breakthrough SVT episodes occurred during the NPO period on the 3 mg/kg/day rectal dose, and no bradycardia, hypoglycemia, or hemodynamic adverse event attributable to propranolol was observed.

**Fig. 2:**
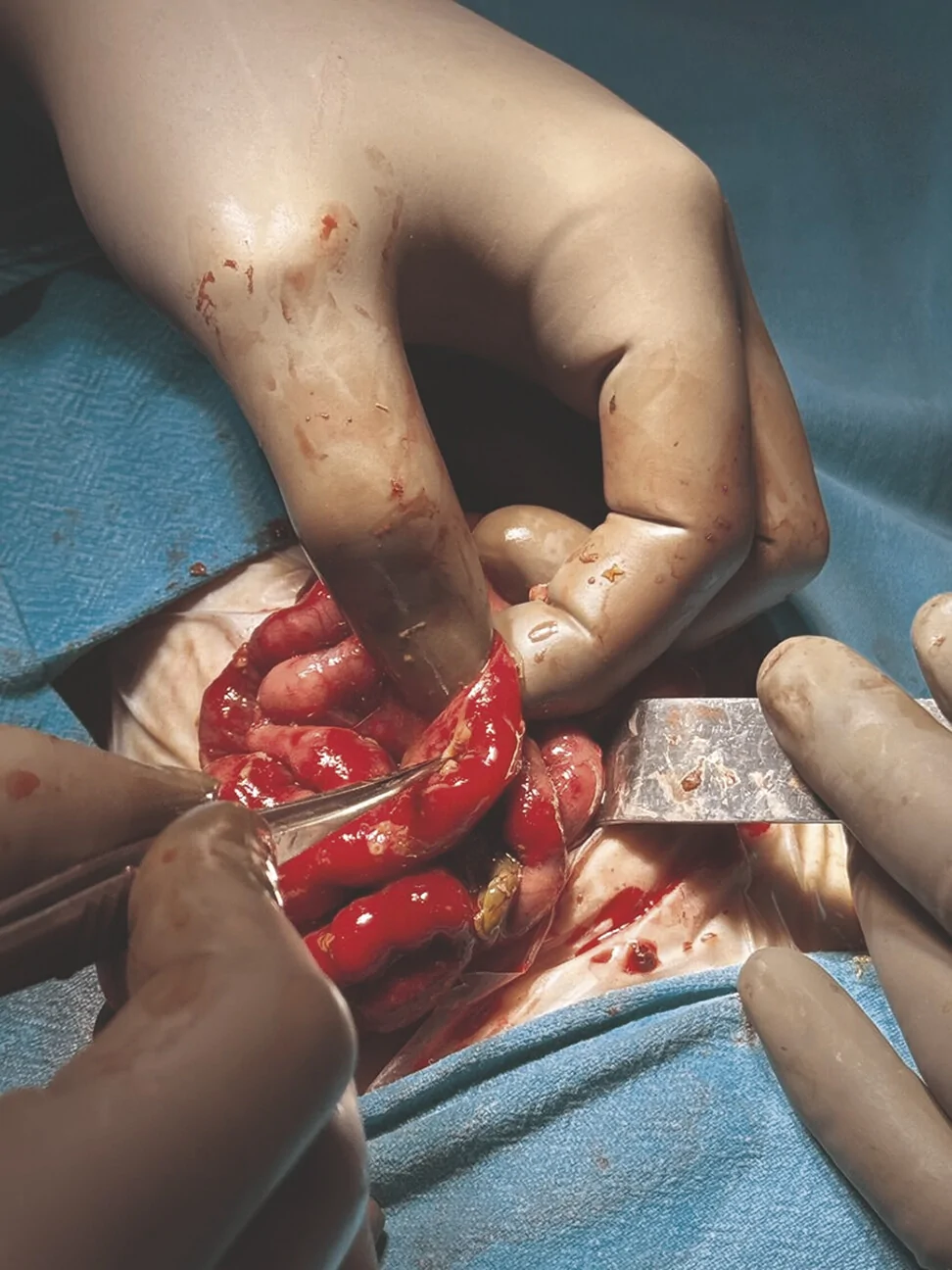
Intraoperative photograph at exploratory laparotomy (DOL 9) demonstrating grossly congested, oedematous, and hemorrhagic small bowel loops with multiple perforations. Surgical retractors are in situ. Two-site resection and anastomosis were performed: jejuno–jejunal and ileo–caecal.

Despite maximal medical management, the infant exhibited progressive clinical deterioration. On DOL 8, repeat abdominal radiography demonstrated pneumoperitoneum ( Fig. 1B ), upgrading the diagnosis to Bell stage 3B NEC. Pediatric surgical consultation was obtained, and a gloved drain was inserted, yielding a mixture of meconium and serous fluid. Operative intervention was undertaken on DOL 9. At laparotomy via a right horizontal incision, multiple intestinal perforations were identified ( Fig. 2 ). Two-site resection and anastomosis were performed: (1) jejuno–jejunal anastomosis (a small perforation in the proximal jejunum and peritoneal soiling were noted) and (2) ileo–caecal resection and anastomosis with closure of multiple smaller perforations. The procedure was uncomplicated. The postoperative period was managed in the NICU with continued mechanical ventilation and inotropic support, which were weaned and withdrawn by postoperative days 2 and 3, respectively. Histopathological examination of the resected terminal ileum and ileo–caecal junction demonstrated marked acute-on-chronic suppurative necrotizing inflammation with serositis, and reactive changes in associated mesenteric lymph nodes, confirming NEC.

A dye study performed on postoperative day 5 (DOL 14) confirmed anastomotic integrity without leakage, and drains were removed. Enteral feeding was reintroduced incrementally from DOL 16. On DOL 16, coincident with the commencement of MEN, an attempt was made to transition propranolol back to the oral route. Within this period, the infant experienced a breakthrough SVT episode. The per-rectal route was therefore reinstated and the dose uptitrated to 4 mg/kg/day rectally (three divided doses), which promptly restored and maintained sinus rhythm. The per-rectal route was continued at 4 mg/kg/day until full enteral feeds were established. Oral feeds were successfully established by DOL 19, at which point propranolol was transitioned to oral administration at 4 mg/kg/day in three divided doses without further SVT recurrence. The infant was transitioned to ad libitum breastfeeding with human milk fortifier supplementation. The infant was discharged on DOL 25 at a corrected gestational age of 38 ^3/7^ weeks, on oral propranolol (4 mg/kg/day in three divided doses), cholecalciferol, and calcium supplementation.

Discharge planning included providing the parents with a portable pulse oximeter, parental training in cardiopulmonary resuscitation, and provision of adenosine vials and written instructions for emergency use at the nearest facility.

## Discussion

We report a case of Bell stage 3B NEC requiring surgical intervention in a late preterm neonate with antenatal-onset SVT, a combination that has not been previously described in the published literature from India. While the association between SVT and NEC has been reported across approximately ten cases since 1995, nearly all prior reports describe Bell stage 1 or 2 NEC managed conservatively with bowel rest and antibiotics. 6 7 8 9 10 11 12 13 14 15 Only the seminal case described by Hajivassiliou and Pitkin in 1998 and the case by Hanna in 2013 required laparotomy, and neither involved antenatal arrhythmia of this severity. 7 9 By contrast, our patient progressed to pneumoperitoneum within 4 days of NEC onset, necessitating surgical intervention with two-site intestinal resection.

The pathophysiological link between SVT and NEC is hypothesized to operate through mesenteric hypoperfusion. During SVT, the shortened diastolic filling time and high heart rate reduce stroke volume and cardiac output. The “steal phenomenon,” mediated by increased systemic vascular resistance, preferentially redistributes blood flow away from the splanchnic bed, resulting in mesenteric ischemia. 10 11 When sinus rhythm is restored either spontaneously or via cardioversion, reperfusion injury may compound the ischemic insult through generation of reactive oxygen species and activation of the proinflammatory cascade (tumor necrosis factor-α, interleukins, prostaglandins, leukotrienes, and complement fragments). 12 This injury pattern closely mirrors the established immunopathogenesis of NEC.

In our case, the antenatal fetal tachyarrhythmia (heart rate > 300 bpm) preceded delivery, suggesting that mesenteric hypoperfusion may have begun in utero, rendering the intestinal mucosa ischemic prior to extrauterine life. This is conceptually supported by the atrial flutter case reported by Tandircioglu et al, in whom repeated cardioversion for antenatally diagnosed arrhythmia was temporally linked to NEC onset. 15 The additional hemodynamic stress of neonatal transition, prematurity, and early respiratory distress syndrome in our patient likely further compromised splanchnic perfusion in the first days of life, culminating in the explosive onset of stage 3B NEC by DOL 4–8. A brief comment on the antenatal decision-making is warranted. Transplacental antiarrhythmic therapy with agents such as digoxin, flecainide, or sotalol was not attempted prior to delivery in this case. The decision to proceed immediately to emergency caesarean section was driven by several concurrent obstetric indications independent of the arrhythmia: a previous uterine scar with scar tenderness and radiological evidence of thinning, active labor, and a nonreassuring nonstress test. The fetal tachyarrhythmia was identified incidentally on the preoperative scan, rather than being the primary indication for delivery. Furthermore, the absence of fetal hydrops or echocardiographic features of congestive cardiac failure on any prior or current scan suggested that the arrhythmia was likely of short duration, reducing the urgency and feasibility of transplacental pharmacological intervention. This is clinically important context: in cases where fetal tachyarrhythmia is detected earlier in gestation without independent obstetric indications for delivery, transplacental antiarrhythmic therapy should be considered, as it may reduce the duration of mesenteric hypoperfusion and thereby potentially mitigate the risk of subsequent NEC.

The only controlled analytical study to date, a single-center case-control study by Jimale et al involving 74 NEC cases and 74 matched controls, found no statistically significant association between SVT and NEC ( *p* = 1.00), noting that no infant in either group had a history of SVT. 16 This null result, however, may reflect the rarity of SVT in unselected NICU populations and the consequent low statistical power, rather than a true absence of association. The authors called for multicenter studies, a recommendation we reiterate.

This case is also, to the best of our knowledge, the first reported case of SVT-associated surgical NEC from India. Although NEC is well-recognized in Indian NICUs, 17 its co-occurrence with neonatal SVT has not been previously documented in the Indian literature. This report extends the literature geographically and emphasizes the importance of heightened clinical vigilance for NEC in any neonate with SVT, regardless of hemodynamic stability.

A further novel aspect of this case is the management of concurrent SVT prophylaxis during the mandatory NPO period imposed by NEC. This clinical dilemma as to how to maintain arrhythmia prophylaxis when both oral and enteral routes are contraindicated has received surprisingly little attention in the literature. Saini et al encountered this problem in both of their cases, reporting that switching from oral to intravenous propranolol resulted in clinically significant hypotension in one infant, necessitating a change to intravenous esmolol. 10 Intravenous esmolol, while titratable and short-acting, requires continuous infusion and may be unavailable or hemodynamically unsafe in critically ill neonates already requiring vasopressor support. In our patient, who was inotrope-dependent with refractory shock, we instead administered propranolol via the per-rectal route using the existing oral liquid preparation at the same mg/kg dosing. This decision was supported by published pharmacokinetic literature demonstrating that propranolol administered into the lower rectum partially bypasses hepatic first-pass metabolism: unlike the superior rectal vein (which drains via the inferior mesenteric vein into the portal circulation), the inferior and middle rectal veins drain directly into the systemic circulation via the inferior vena cava. 18 19 Importantly, rectal absorption in neonates is generally increased relative to older children and adults, owing to higher rectal mucosal blood flow and reduced mucosal surface area, though variability in the depth of insertion may affect dose consistency. 20 In our case, this strategy was clinically effective: no SVT recurrence occurred during the NPO period on a rectal propranolol dose of 3 mg/kg/day. Critically, when an attempt was made to transition back to oral propranolol on DOL 16 coincident with the introduction of MEN, a breakthrough SVT episode occurred. The per-rectal route was reinstated and the dose uptitrated to 4 mg/kg/day rectally, which promptly restored sinus rhythm; this was maintained until full enteral feeds were established on DOL 19, at which point oral propranolol at 4 mg/kg/day was re-introduced without further SVT recurrence. This sequence of SVT on oral withdrawal, resolution on rectal reinstatement and uptitration, perhaps, constitutes direct pharmacological evidence of rectal propranolol efficacy. It also demonstrates that the rectal route is titratable, with dose escalation achievable in a manner analogous to oral administration. No hemodynamic adverse event attributable to propranolol was observed throughout. To the best of our knowledge, this is the first description of per-rectal propranolol as an antiarrhythmic bridging strategy in a neonate, representing a practical, low-cost, and immediately available solution to an otherwise unresolved management dilemma in the co-occurrence of SVT and surgical NEC. A systematic search of PubMed and Medline using the terms “propranolol,” “rectal,” and “neonate” or “infant” or “newborn” identified no prior report of rectal propranolol used as an antiarrhythmic agent in this age group, supporting the novelty of this approach.

Table 1 presents a comprehensive synthesis of all cases of neonatal SVT-associated NEC reported in the literature, including the present case. The progression from early case reports involving term infants with congenital heart disease to predominantly preterm and late-preterm infants without structural cardiac abnormalities reflects an evolving understanding of this association.

**Table 1:** Published cases of neonatal supraventricular tachyarrhythmia associated with NEC.

Author (y)	GA/BW	SVT Onset	HR (bpm)	NEC onset/presentation	Imaging	Treatment	Notes
Khalak and Chess (1998) 8	29 wk/NR	Antenatal	NR	DOL 4—fulminant NEC	NR	Parenteral nutrition; antibiotics	Fatal; no CHF signs
Hajivassiliou and Pitkin (1998) 7	41 wk/NR	1 mo of life	NR (refractory)	Month 5—abdominal distension	X-ray: no pneumatosis; features of paralytic ileus	Surgery (hemicolectomy); antibiotics; TPN	Tricuspid atresia; B–T shunt; recurrent NEC
Cale and Speidel (1995) 6 , ^a^	Premature/NR	Antenatal	NR	DOL 8	Extensive NEC on imaging	Not detailed	First reported case; cited in Hanna 9
Hanna (2013) 9	35 wk/2200 g	Antenatal	240–250	DOL 11—emesis, distension	X-ray: pneumatosis	Surgery (bowel resection); antibiotics; TPN	No CHD; SVT controlled on propranolol/amiodarone
Saini et al (2017)—case 1 10	36 wk/2100 g (IUGR)	DOL 3	280	DOL 7—rectal bleeding, distension	Ultrasound: pneumatosis; X-ray negative	Antibiotics (ampicillin, tobramycin, metronidazole); TPN	Miller–Dieker syndrome; PFO + small PDA
Saini et al (2017)—Case 2 10	36 wk/LGA	DOL 1	250	DOL 7—bloody stools	X-ray + ultrasound: colonic pneumatosis	Antibiotics (vancomycin, tobramycin, metronidazole); TPN	Wolff–Parkinson–White; fenestrated atrial septum + rPDA
Nakib et al (2018) 12	38 wk/2755 g	DOL 13	278–294	DOL 16/37—distension; obstruction	Contrast enema: transverse colon stenosis	Surgery (resection + anastomosis)	First term infant without CHD; histology confirmed ischemia
Mecarini et al (2020) 11	29 ^6/7^ wk/1380 g	DOL 16	300–390	DOL 17—bloody stools, distension	X-ray: no pneumatosis; ultrasound: normal	Antibiotics (ampicillin + gentamicin); TPN; amiodarone switch	Twin-to-twin transfusion; managed conservatively
Tandircioglu et al (2022) 15	37 wk/3000 g	Antenatal (35 wk)	180 (ventricular 4:1)	DOL 2 (16 h postcardioversion)—bloody stool, distension	X-ray: no pneumatosis; thrombocytopenia	Antibiotics; NPO; discharged on amiodarone + LMWH	Atrial flutter; NEC precipitated by repeated cardioversion
This case (2026)	35 wk/2740 g	DOL 3	280–300	DOL 4—hematochezia, distension; DOL 8 pneumoperitoneum (stage 3B)	X-ray: pneumatosis + portal gas → pneumoperitoneum; ultrasound: ascites	Surgery (jejuno–jejunal + ileo–caecal resection-anastomosis); meropenem + metronidazole; TPN; propranolol	Fetal tachyarrhythmia >300 bpm; ASD; refractory shock; HPE confirmed NEC with serositis; per-rectal propranolol used during NPO—first reported neonatal use

Abbreviations: ASD, atrial septal defect; BW, birth weight; CHD, congenital heart disease; DOL, day of life; GA, gestational age; IUGR, intrauterine growth restriction; LGA, large for gestational age; LMWH, low molecular weight heparin; NEC, necrotizing enterocolitis; NR, not reported; PDA, patent ductus arteriosus; PFO, patent foramen ovale; SVT, supraventricular tachycardia; TPN, total parenteral nutrition.

^a^ Reported as a letter; full data not available.

Several limitations of this report merit explicit acknowledgment. First, plasma propranolol levels were not measured during the per-rectal dosing period, and therefore bioavailability of the rectal preparation cannot be confirmed quantitatively. However, the concern that sinus rhythm during the NPO period may have reflected preexisting rhythm stability rather than drug effect is substantially mitigated by a key clinical observation: on DOL 16, when an attempt was made to transition to oral propranolol coincident with the commencement of MEN, a breakthrough SVT episode occurred. Reinstatement of the per-rectal route with dose uptitration to 4 mg/kg/day promptly restored and maintained sinus rhythm through to full enteral feeds on DOL 19, at which point oral propranolol was successfully re-established without further recurrence. This sequence provides direct clinical evidence that per-rectal propranolol was very likely pharmacologically active, therapeutically effective, and titratable. Second, neonatal rectal bioavailability of propranolol is subject to known inter-individual variability, 20 related to differences in depth of drug insertion, rectal mucosal blood flow, and concurrent pathophysiological states including hemodynamic compromise. Third, as a single case report, definitive conclusions regarding the safety and efficacy of rectal propranolol as a routine antiarrhythmic bridging strategy cannot be drawn; formal pharmacokinetic studies and additional clinical experience in neonates are required before this approach can be recommended as standard practice.

## Conclusion

This case of Bell stage 3B surgical NEC in a late preterm neonate with antenatal SVT highlights the potentially catastrophic consequences of SVT-induced mesenteric ischemia and extends the published case series geographically to India. Neonatologists and pediatric cardiologists should maintain a high index of suspicion for NEC in any neonate presenting with SVT, initiate early serial abdominal surveillance (clinical, radiographic, and ultrasonographic), and promptly involve pediatric surgery when clinical or radiological signs of bowel necrosis emerge. The concurrent management of SVT prophylaxis and mandatory NPO status for NEC presents a clinically challenging dilemma; per-rectal propranolol, leveraging its partial avoidance of hepatic first-pass metabolism via the inferior rectal venous drainage, represents a novel, practical, and effective bridging strategy that deserves further investigation. Multicenter, prospective data collection is essential to quantify the risk of NEC among neonates with SVT and to identify both clinical predictors of severe NEC and optimal antiarrhythmic strategies in this unique population.
